# The Effect of Sex, Race/Ethnicity, and Neighborhood Socioeconomic Disadvantage on Total Hip Arthroplasty Utilization: A Multicenter Cohort Study

**DOI:** 10.5435/JAAOSGlobal-D-25-00054

**Published:** 2025-08-05

**Authors:** Katherine A. Woolley, Nicole J. Newman-Hung, Mikayla Mefford, Charlotte F. Wahle, Emma D. Grellinger, Chloe Dlott, Hannah Chi, Nicholas J. Jackson, Stephanie E. Wong, Lauren E. Wessel, Alexandra I. Stavrakis

**Affiliations:** From the Department of Orthopaedic Surgery, University of California, San Francisco, CA (Dr. Woolley, Dr. Dlott, and Dr. Wong); the Department of Orthopaedic Surgery, University of California, Los Angeles, CA(Dr. Newman-Hung, Dr. Wessel, and Dr. Stavrakis); the David Geffen School of Medicine, University of California, Los Angeles, CA (Ms. Mefford, and Ms. Wahle); School of Medicine, the University of California, San Francisco, CA (Ms. Grellinger and Dr. Chi); and the Division of General Internal Medicine and Health Services Research, University of California, Los Angeles, CA (Dr. Jackson)

## Abstract

**Introduction:**

Disparities in the management of hip osteoarthritis (OA) exist across sex, race/ethnicity, and socioeconomic status, but their combined effect on total hip arthroplasty (THA) utilization remains unclear. This study evaluates differences in presentation, nonsurgical treatments, and THA rates across two academic centers.

**Methods:**

Patients with primary hip OA seen in 2002 at two tertiary academic centers were included. Demographics, Kellgren-Lawrence grade, Charlson Comorbidity Index (CCI), and nonsurgical treatments were collected. Socioeconomic status was assessed using the social deprivation index, stratified into quartiles (Q4 = most deprived). Chi square, analysis of variance, and *t*-tests compared demographics and utilization rates. A multivariable model analyzed factors influencing THA likelihood.

**Results:**

Among 456 patients, 328 were recommended for THA for primary hip OA, 63% were female, 6% were Asian, 6% Black, 23% other, 3% unknown, 54% White, and 8% Hispanic. Female patients were older (70.3 ± 8.6 vs. 68.1 ± 9.7 years, *P* = 0.03). Hispanic patients were younger (62.2 ± 12.1 vs. 70.1 ± 8.7 years, *P* = 0.0020) with lower CCI (2.20 ± 1.52 vs. 2.93 ± 1.49 vs. 3.28 ± 1.69 *P* = 0.027). Social deprivation index Q4 patients had greater physical therapy utilization (Q4 79% vs. Q1 62%, *P* = 0.006). Overall, 79% of patients who were offered THA underwent surgery, with multivariate analysis revealing lower likelihood among females, Black, Asian, and Hispanic patients with higher CCI (*P* < 0.001), whereas socioeconomically disadvantaged patients were more likely to undergo THA (*P* < 0.05).

**Conclusion:**

Although THA utilization was high, disparities in presentation age, nonsurgical treatments, and comorbidities suggest differing challenges across populations. Future research should explore drivers of these disparities.

**Level of Evidence:**

IV retrospective cohort

Osteoarthritis (OA) is increasing in frequency, and more patients will benefit from total hip arthroplasty (THA) if they are able to access timely and effective care.^1^ However, disparities in the management of hip OA and THA utilization have been documented well, with evidence showing that sex, race/ethnicity, and socioeconomic factors influence access to care and surgical outcomes, including length of stay, readmissions, and emergency room visits.^2–6^ Prior research has demonstrated that women are less likely to undergo surgery for OA and often undergo surgery at older ages.^4,5,7^ Patients from racial and ethnic minority backgrounds are also less likely to undergo THA and experience more complications following surgery.^2,8–11^ Patients with lower socioeconomic status (SES) have also been found to undergo THA at lower rates than those with higher SES and have increased complication rates following surgery.^8,12,13^ However, the combined effect of these demographic variables and socioeconomic disadvantage at the institutional level remains unclear.

In addition, prior investigations have focused on national registry data as opposed to institutional level analyses. This multicenter cohort study primarily aimed to evaluate differences in THA utilization rates based on sex, race/ethnicity, and neighborhood socioeconomic disadvantage across two tertiary academic centers. Secondary objectives included assessing disparities in presenting symptomatology, nonsurgical treatment courses, and radiographic OA severity.

## Methods

A database of THA candidates who were seen for initial consultation at two tertiary academic centers in the calendar year 2022 was created. Patient demographics, Kellgren-Lawrence (KL) grade, duration of symptoms from onset to presentation to arthroplasty clinic, visual analogue scale, Charlson Comorbidity Index (CCI), and nonsurgical treatment modalities trialed (nonsteroidal anti-inflammatory medications, opioid medications, corticosteroid or viscosupplementation injections, physical therapy, and bracing treatment) and zip code were collected. Socioeconomic disadvantage was assessed using the social deprivation index (SDI), which uses census-tract level information from the American Community Survey on poverty, education, employment, and family structure to create an aggregate score. Values range from 0 to 100, with higher values indicating greater neighborhood-level social disadvantage. To link these census-tract measures with our patient data, SDI values were averaged by zip code.

Patients who were offered surgery, with severe or KL 3 to 4 primary OA were included. Patients with mild or KL grade 1 to 2 OA or diagnoses other than primary OA (i.e., posttraumatic arthritis, inflammatory arthritis, osteonecrosis); hemiarthroplasty, nonelective arthroplasty (i.e., THA for femoral neck fracture); revision arthroplasty; and patients not offered surgery were excluded.

Presenting characteristics and utilization rates of THA were compared between patient groups based on demographics (i.e., sex, race, ethnicity) using chi square, analysis of variance, and Welch *t*-test. A multivariable logistic regression model was used to understand factors that were associated with undergoing THA. Variable selection was based on the model by Hartnett et al 1^14^ examining socioeconomic disparities in THA, which included age, sex, race, ethnicity, SDI, and CCI. Insurance information was not collected and thus not included in our multivariable model. Clustered robust standard errors were used to account for correlated outcomes among sites. All analyses were conducted in Stata version 18.1, Stata.

## Results

Four hundred fifty-six new patients underwent consultation at both institutions in 2022. Three hundred twenty-eight were offered THA for primary hip OA and met inclusion criteria. Two hundred seven (63%) were female, and 121 (37%) were male. Overall, there were 177 White (54%), 29 Asian (6%), 21 Black (6%), 25 Hispanic (8%), 76 other race (23%), and 10 unknown race (3%) patients.

Female patients were markedly older at initial presentation than male patients (70.3 ± 8.6 vs. 68.1 ± 9.7 years; *P* = 0.03; Table 1). Hispanic patients presented at markedly younger ages compared with non-Hispanic patients (62.2 ± 12.1 vs. 70.1 ± 8.7 years; *P* = 0.0020) and had lower comorbidity scores (2.20 ± 1.52 vs. 2.93 ± 1.49 vs. 3.28 ± 1.69; *P* = 0.027). No differences were found in symptom duration, KL grade, or nonsurgical treatments (Table 2).

**Table 1:** Demographic Data and Surgical Utilization Rates by Sex

Variable	Total (n)	Females (n)	Males (n)	*P*
Age (years; mean ± SD)	69.5 ± 9.1	(207) 70.3 ± 8.6	(121) 68.1 ± 9.7	0.03^a^
CCI	3.05 ± 1.61	3.11 ± 1.59	2.96 ± 1.64	0.49
Surgery	(270) 79%	(167) 78%	(103) 79%	0.89
KL 3	(63) 26%	(38) 27%	(25) 25%	0.88
KL 4	(177) 74%	(103) 73%	(74) 75%	
NSAIDs	(146) 61%	(89) 64%	(57) 58%	0.42
Opioids	(19) 8%	(11) 8%	(8) 8%	1.00
Injections	(78) 33%	(46) 33%	(32) 33%	1.00
PT	(157) 66%	(95) 67%	(62) 63%	0.58
Duration of symptoms (mo) median (IQR)	24.0 [12.0-36.0]	24.0 [12.0-36.0]	24.0 [9.0-36.0]	0.81
SDI (mean ± SD)	8.84 ± 11.05	9.34 ± 11.71	7.98 ± 9.81	0.26

CCI = Charlson Comorbidity Index, IQR = interquartile range, KL = Kellgren-Lawrence, NSAIDs = nonsurgical treatment modalities, PT = prothrombin time, SDI = social deprivation index

a Statistical significance.

**Table 2:** Demographic Data and Surgical Utilization Rates by Ethnicity

Factor or Variable	Non-Hispanic Non-White	White	Hispanic	*P*
Age (years; mean ± SD)	68.8 ± 8.7	71.0 ± 8.4	62.2 ± 12.1	<0.001^a^
CCI	2.93 ± 1.49	3.28 ± 1.69	2.20 ± 1.52	0.027^a^
Surgery	(100) 76%	(149) 80%	(21) 81%	0.70
KL 3	(32) 28%	(27) 25%	(4) 25%	0.83
KL 4	(82) 72%	(83) 75%	(12) 75%	
NSAIDs	(70) 62%	(68) 61%	(8) 53%	0.79
Opioids	(7) 6%	(10) 9%	(2) 13%	0.55
Injections	(40) 36%	(32) 29%	(6) 40%	0.45
PT	(80) 71%	(67) 60%	(10) 67%	0.26
Duration of symptoms (mo) median [IQR]	18.0 [10.0-36.0]	30.0 [12.0-36.0]	24.0 [12.0-36.0]	0.94
SDI (mean ± SD)	9.29 ± 10.16	7.54 ± 10.35	15.84 ± 16.61	0.001^a^

CCI = Charlson Comorbidity Index, IQR = interquartile range, KL = Kellgren-Lawrence, NSAIDs = nonsurgical treatment modalities, PT = prothrombin time, SDI = social deprivation index

a Statistical significance.

**Figure 1:**
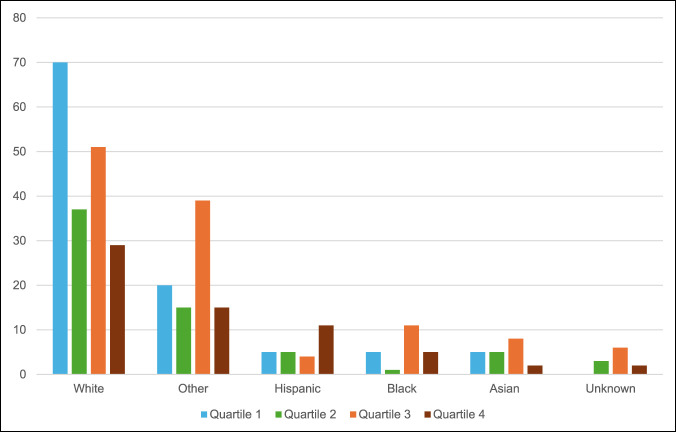
Chart showing social deprivation index quartiles by race.

Patients from the least socioeconomically disadvantaged quartile (SDI Q1) accounted for 30% of the total patient cohort, whereas the most SDI Q4 accounted for 16%. SDI Q1 comprised 26.3% of the Asian patient cohort, 23.8% of the Black patient cohort, 21% of the other patient cohort, 0 unknown race patients, 39% of the White patient cohort, and 20% of the Hispanic patient cohort. SDI Q4 was comprised 5.3% of the Asian patient cohort, 19% of the Black patient cohort, 13.2% of the other patient cohort, 20% of the unknown patient cohort, 13.6% of the White patient cohort, and 44% of the Hispanic patient cohort (Figure 1). Hispanic patients had markedly higher SDI (15.8) compared with non-Hispanic and non-White (9.3) and White patients (7.5; *P* = 0.001; Table 2). Patients from more SDI Q4 neighborhoods were more likely to have used physical therapy before consultation (Q4 79% vs. Q1 62%, *P* = 0.006; Table 3).

**Table 3:** Demographic Data and Surgical Utilization by Social Deprivation Index Quartile

Factor or Variable	Quartile 1	Quartile 2	Quartile 3	Quartile 4	*P*
Age (yr) (mean ± SD)	70.7 ± 9.6	70.4 ± 8.2	67.9 ± 8.7	69.7 ± 10.1	0.13
CCI (mean ± SD)	3.11 ± 1.53	3.02 ± 1.45	3.06 ± 1.87	2.96 ± 1.20	0.98
Surgery	(79) 79%	(45) 74%	(91) 79%	(44) 85%	0.57
KL 3	(20) 30%	(15) 33%	(24) 26%	(3) 11%	
KL 4	(46) 70%	(30) 67%	(68) 74%	(25) 89%	0.16
NSAIDs	(40) 62%	(22) 50%	(60) 66%	(18) 62%	0.36
Opioids	(5) 8%	(4) 9%	(6) 7%	(4) 14%	0.67
Injections	(19) 29%	(17) 39%	(30) 33%	(12) 41%	0.61
PT	(41) 62%	(21) 48%	(68) 75%	(23) 79%	0.0006^a^
Symptom duration (mo) median [IQR]	24.0 [7.0-36.0]	24.0 [11.0-36.0]	30.0 [12.0-36.0]	30.0 [12.0-48.0]	0.61

CCI = Charlson Comorbidity Index, IQR = interquartile range, KL = Kellgren-Lawrence, NSAIDs = nonsurgical treatment modalities, PT = prothrombin time

a Statistical significance.

Overall, 270 patients (79%) who were offered THA underwent surgery and 58 (18%) did not. No differences were found in utilization rates by sex, race/ethnicity, or SDI quartile on univariate analysis. A multivariate model was used to investigate any additional associations with increased or decreased likelihood of undergoing THA. After controlling for factors affecting utilization outlined in the prior literature, we found that female patients were less likely to undergo THA (OR 0.78, *P* < 0.001). Patients with higher CCI were also less likely to undergo THA (OR 0.72, *P* < 0.001). In addition, with White as the reference race, Asian, Black, and Hispanic patients were also less likely to undergo THA (OR 0.52, OR 0.47, OR 0.28, *P* < 0.001). Interestingly, patients with increased SDI or those with worse socioeconomic disadvantage were more likely to undergo surgery (OR 1.01, *P* = 0.01). Finally, patients of other race were also more likely to undergo THA (OR 2.4, *P* < 0.001; Table 4).

**Table 4:** Multivariate Model of Odds of Surgical Utilization

Factor or Variable	OR	*P*
Age	0.981 (0.951-1.012)	0.23
Female	0.776 (0.748-0.805)	<0.001^a^
Asian	0.523 (0.493-0.554)	<0.002^a^
Black	0.469 (0.460-0.478)	<0.003^a^
Other race	2.407 (1.940-2.985)	<0.004^a^
Unknown race	0.321 (0.063-1.632)	0.17
Hispanic	0.278 (0.156-0.494)	<0.001^a^
SDI	1.006 (1.001-1.011)	0.01^a^
CCI	0.724 (0.675-0.777)	<0.001^a^

CCI = Charlson Comorbidity Index, SDI = social deprivation index

a Statistical significance.

## Discussion

Disparities in the management of hip OA and THA utilization are well documented with prior research demonstrating that sex, race/ethnicity, and socioeconomic factors influence access to care and surgical outcomes, such as postoperative length of stay, readmission rates, and overall complications.^2,9–11^ Given the prevalence of these disparities, we sought to further investigate whether differences in THA utilization rates based on sex, race/ethnicity, and neighborhood socioeconomic disadvantage persisted across two tertiary academic centers.

Our study found high THA utilization rates across groups; however, differences in age at presentation, nonsurgical treatment allocation, and comorbidity burden persisted. Female patients presented for initial consultation at older ages while Hispanic patients were nearly 10 years younger at the time of presentation and surgery compared with non-Hispanic patients. In addition, like prior research, we found that female and non-white patients were less likely to undergo THA.^2,10^ Interestingly, patients with increased socioeconomic disadvantage were slightly more likely to undergo surgery, which may reflect the influence of academic center practices that prioritize reducing barriers to care.

There are conflicting studies in the literature regarding differences in THA utilization among women. Some studies demonstrate that women are less likely to be offered THA than men and are less likely to undergo THA.^4,7^ When women undergo THA, they are more likely to be older, which is similar to our findings.^5^ Woolley et al^15^ found no difference in utilization of THA based on sex in a single institution cohort. In our multivariable model, women were less likely to undergo THA than men. Although women have a higher prevalence of OA, there may be complex social situations that lead to later presentation for THA including availability of support at home following surgery.^5,7,16^

Similar to prior research, our multivariable analysis found that patients who identify as Asian, Black, or Hispanic were less likely to undergo THA. A systematic review by Alvarez et al^17^ included 82 articles and found that compared with White patients, Black, Hispanic, and Asian patients were less likely to undergo THA. This could be due to a variety of factors including patient expectations, cultural differences, or difficulty accessing care for THA. Over time, there have been improvements in utilization for Black patients, but disparities persist.^18^

In contrast to prior investigations, our study found that patient with lower SES were more likely to undergo THA and to have participated in prothrombin time before undergoing THA. Dlott et al^12^ found that patients with lower net worth were underusing THA compared with patients with higher net worths, although our study used home address as a proxy for SES as opposed to net worth. For patients undergoing rotator cuff repair, Paul et al^19^ found that patients with lower SES were less likely to undergo rotator cuff repair or participate in physical therapy. Potential reasons for increased utilization of THA in our cohort for patients with lower SES could be due to academic institutions providing a central hub of specialists, which may reduce barriers to care for underserved populations who may lack access to surgical consultation. In addition, academic centers may be more likely to be near urban centers, accept Medicaid, and have streamlined referral systems.

Addressing disparities in THA utilization for patients is essential to ensure equitable access to health care. However, understanding patient's rationale for pursuing THA is critical to ensure that patients receive personalized patient counseling and postoperative care.^15,20^ Amen et al^21^ demonstrated that time to surgery is similar between patients once they decide to proceed, so disparities existing before this decision point may be mitigated by improved patient-specific counseling.

One limitation of this study is the retrospective study design; however, we feel that this study provides proof of concept and lays the groundwork for further studies that could include more study locations and use a prospective design. With any retrospective study, there is the potential for selection bias, which we mitigated by using strict inclusion and exclusion criteria. Another potential limitation is that this study was conducted at two academic tertiary care centers, which may make the results limited to these practice settings. However, given patients seek care are tertiary care centers, it is important to investigate the disparities that may exist in these locations.

## Conclusion

Although THA utilization rates were high across groups in our patient cohort, disparities in age at presentation, nonsurgical treatment allocation, and comorbidity burden highlight different challenges faced by certain populations. Multivariate analysis followed national trends indicating that female and non-White patients are less likely to undergo THA. In contrast to prior research, patients with increased socioeconomic disadvantage were more likely to undergo surgery, which may reflect the influence of academic center practices that prioritize reducing barriers to care. Future research should explore factors driving differences in presenting characteristics, nonsurgical treatment courses, and trends in THA utilization. In addition, future studies should be prospective and include more geographic variability.

## References

[1] Sloan M Premkumar A Sheth NP: Projected volume of primary total joint arthroplasty in the U.S., 2014 to 2030. J Bone Joint Surg Am 2018;100:1455-1460. 30180053 10.2106/JBJS.17.01617

[2] Amen TB Varady NH Rajaee S Chen AF: Persistent racial disparities in utilization rates and perioperative metrics in total joint arthroplasty in the U.S.: A comprehensive analysis of trends from 2006 to 2015. J Bone Joint Surg Am 2020;102:811-820. 32379122 10.2106/JBJS.19.01194

[3] Weiner JA Adhia AH Feinglass JM Suleiman LI: Disparities in hip arthroplasty outcomes: Results of a statewide hospital registry from 2016 to 2018. J Arthroplasty 2020;35:1776-1783.e1. 32241650 10.1016/j.arth.2020.02.051

[4] O’Connor MI: Sex differences in osteoarthritis of the hip and knee. J Am Acad Orthop Surg 2007;15:S22-S25. 17766785

[5] Cheah C Hussein IH El Othmani A Rizvi SA Sayeed Z El-Othmani MM: Assessing preoperative risk factors with sex disparities in total joint arthroplasty patients and financial outcomes from the national inpatient sample database. J Am Acad Orthop Surg 2020;28:e969-e976. 32015251 10.5435/JAAOS-D-19-00716

[6] SooHoo NF Farng E Lieberman JR Chambers L Zingmond DS: Factors that predict short-term complication rates after total hip arthroplasty. Clin Orthop Relat Res 2010;468:2363-2371. 20428982 10.1007/s11999-010-1354-0 PMC2914297

[7] Hawker GA Wright JG Coyte PC : Differences between men and women in the rate of use of hip and knee arthroplasty. N Engl J Med 2000;342:1016-1022. 10749964 10.1056/NEJM200004063421405

[8] Alvarez PM McKeon JF Spitzer AI : Socioeconomic factors affecting outcomes in total knee and hip arthroplasty: A systematic review on healthcare disparities. Arthroplasty 2022;4:36. 36184658 10.1186/s42836-022-00137-4 PMC9528115

[9] Okike K Chan PH Prentice HA Navarro RA Hinman AD Paxton EW: Association of race and ethnicity with total hip arthroplasty outcomes in a universally insured population. J Bone Joint Surg Am 2019;101:1160-1167. 31274717 10.2106/JBJS.18.01316

[10] Shahid H Singh JA: Racial/ethnic disparity in rates and outcomes of total joint arthroplasty. Curr Rheumatol Rep 2016;18:20. 26984804 10.1007/s11926-016-0570-3

[11] Stone AH MacDonald JH Joshi MS King PJ: Differences in perioperative outcomes and complications between African American and white patients after total joint arthroplasty. J Arthroplasty 2019;34:656-662. 30674420 10.1016/j.arth.2018.12.032

[12] Dlott CC Pei X Ittner JL Lefar SL O'Connor MI: Intersectionality of net worth and race relative to utilization of total hip and knee arthroplasty. J Arthroplasty 2021;36:3060-3066.e1. 34099350 10.1016/j.arth.2021.04.037

[13] Agabiti N Picciotto S Cesaroni G ; Italian Study Group on Inequalities in Health Care: The influence of socioeconomic status on utilization and outcomes of elective total hip replacement: A multicity population-based longitudinal study. Int J Qual Health Care 2007;19:37-44. 17159197 10.1093/intqhc/mzl065

[14] Hartnett DA Brodeur PG Kosinski LR Cruz AI Gil JA Cohen EM: Socioeconomic disparities in the utilization of total hip arthroplasty. J Arthroplasty 2022;37:213-218.e1. 34748913 10.1016/j.arth.2021.10.021

[15] Woolley KA Chi H Allahabadi S : Sex-based differences in the utilization of shoulder, hip, and knee arthroplasty. J Am Acad Orthop Surg Glob Res Rev 2023;7:e23.00022. 10.5435/JAAOSGlobal-D-23-00022 PMC10586858 37549367

[16] Mota REM Tarricone R Ciani O Bridges JFP Drummond M: Determinants of demand for total hip and knee arthroplasty: A systematic literature review. BMC Health Serv Res 2012;12:225. 22846144 10.1186/1472-6963-12-225 PMC3483199

[17] Alvarez PM McKeon JF Spitzer AI : Race, utilization, and outcomes in total hip and knee arthroplasty: A systematic review on health-care disparities. JBJS Rev 2022;10. 10.2106/JBJS.RVW.21.00161 35231001

[18] Sheth M Chambers M Gronbeck C Harrington MA Halawi MJ: Total hip arthroplasty in Black/African American patients: An updated nationwide analysis. J Racial Ethn Health Disparities 2021;8:698-703. 32725608 10.1007/s40615-020-00829-0

[19] Paul RW Osman A Nigro A : The effects of social determinants of health on rotator cuff repair utilization and outcomes: A systematic review. JSES Rev Rep Tech 2024;4:346-352. 39157253 10.1016/j.xrrt.2024.03.015 PMC11329048

[20] Adu Y Hurley J Ring D: Are there racial and ethnic variations in patient attitudes toward hip and knee arthroplasty for osteoarthritis? A systematic review. Clin Orthop Relat Res 2024;482:1417-1424. 38393955 10.1097/CORR.0000000000003021 PMC11272247

[21] Amen TB Liimakka AP Jain B Rudisill SS Bedair HS Chen AF: Total joint arthroplasty utilization after orthopaedic surgery referral: Identifying disparities along the care pathway. J Arthroplasty 2023;38:424-430. 36150431 10.1016/j.arth.2022.09.020

